# Can Automated Text Messaging Successfully Monitor Antibiotic Adherence for Urban Adolescents and Young Women Managed for Pelvic Inflammatory Disease in the Outpatient Setting

**DOI:** 10.17140/pnnoj-8-135

**Published:** 2022-03-30

**Authors:** Bria Rice, Jamie Perin, Steven Huettner, Arlene Butz, Hasiya E. Yusuf, Maria Trent

**Affiliations:** 1 Department of Internal Medicine, Mayo Clinic, Phoenix, Arizona, USA; 2 Division of Adolescent and Young Adult Medicine, Department of Pediatrics, Johns Hopkins School of Medicine, Baltimore, MD 21287, USA

**Keywords:** Pelvic inflammatory disease, Sexually transmitted infections, Adolescents, Outpatient, Text messaging, Community health nursing

## Abstract

**Objective:**

This study evaluates patient responsiveness to an automated text messaging system for pelvic inflammatory disease (PID) self-care support, and measures the reliability of text-reported adherence.

**Design:**

Patients aged 13–25-years with mild to moderate PID were recruited from urban, academic outpatient settings. Enrolled patients received antibiotics and were randomized into a standard of care or intervention group. During a 14-day treatment period, participants in the intervention arm received a community-based nursing visit and daily text message medication reminders with evening prompts to report the number of doses taken.

**Result:**

Of the 97 participants randomized into the intervention arm, 91 (94%) were eligible for analysis. Most were African American and low income, with a mean age of 18.3 (SD=2.2) years. Participants responded to ~53% (SD=34%) of all dosage inquiry messages. Responsiveness attenuated at approximately 2.2% per day over the treatment period. Ninety-three percent (n=85) of the analyzed intervention arm returned for the two-week follow-up. Despite overall adherence and general responsiveness, text-reported and self-reported medication adherence were not well correlated (r=0.37, *p*<0.001).

**Conclusion:**

Our findings show that text messaging is a feasible strategy for reaching urban adolescents being managed for complicated sexually transmitted infections in outpatient settings. However, patient responsiveness to self-care text messages do vary, limiting the adherence monitoring capacity of this technology. Given the number of unanswered text messages and incomplete text-reported adherence data, additional measures to assess adolescents’ adherence to PID medications are needed in clinical trials.

## INTRODUCTION

Pelvic inflammatory disease (PID) is a reproductive health disorder that disproportionately affects young,^1,2^ and African American women in the United States.^3–5^ Without proper treatment, PID can cause tubal scarring, infertility, ectopic pregnancy, and chronic pelvic pain.^6^ The Centers for Disease Control and Prevention (CDC) guidelines no longer recommend hospitalization for mild to moderate PID^7^ as inpatient treatment is expensive,^8,9^ and offers limited reproductive health benefits over outpatient management.^10^ Despite evidence that youth with PID struggle with adherence to therapy,^11^ most young women with PID continue to be managed in outpatient settings with oral antibiotics^7^ and no additional clinical support.

For adolescents who make up 20% of all new PID cases,^12^ the shift from inpatient to outpatient treatment without additional clinical support may have been shortsighted. Adolescents often engage in high-risk sexual behaviors, like unprotected sex and sexual concurrency that increase exposure to sexually transmitted diseases (STDs) and PID.^6,13^ Once diagnosed, adolescents with PID struggle to adhere to antibiotics and follow-up appointments.^14^ A 2010 randomized controlled trial (RCT) investigated the benefit of a 6-minute interventional video on adolescent’s adherence to PID self-care recommendations. Participants were found to be only 66% adherent to antibiotics, while a mere 16% of control and 36% of intervention groups returned for the recommended 72-hr follow-up visit.^15^

Additionally, poor adherence to therapy increases adolescents’ risk for short- and long-term sequelae.^16,17^ Adolescents with PID are more likely than adult women to develop recurrent PID and subsequent STDs.^18^ Each PID episode substantially increases the risk of tubal infertility and chronic pelvic pain.^16^ Such disparities suggest that youth-focused interventions are needed to improve health outcomes for adolescents and young adult women managed for PID in outpatient settings.

With ~6 million text messages sent each day,^19^ electronic messaging may provide a novel and cost-effective means^20^ to communicate with patients in outpatient settings, and to improve treatment outcomes. Text messages are delivered in real time,^20,21^ making them suitable for promoting health behavior change.^21,22^ Moreover, text messaging is widely accessible among youth,^23–25^ and there is no digital divide for under-represented minorities.^26,27^ In a 2012 text messaging intervention for urban, minority youth on Depo Provera, 92% of participants had unlimited access to text messaging services.^27–29^ Findings from this study and others^30,31^ demonstrate the feasibility of two-way health related text messaging with minority youth, a population that is often difficult to reach outside of healthcare settings. Text message interventions also improved health outcomes and behaviors for patients with diabetes, asthma, and human immunodeficiency virus (HIV) in prior studies.^21,30–32^ A recent RCT by Wolff et al^33^ used text message reminders to increase adolescents’ adherence to recommended PID follow-up appointments. However, no study has evaluated the role automated text messaging may play in monitoring antibiotic adherence in youth with PID.

The Technology Enhanced Community Health Nursing (TECH-N) study is a two arm RCT designed to evaluate how text message reminders and community health nursing visits may improve urban adolescents’ adherence to antibiotics, and reduce the sequelae of PID.^34,35^ Recently published findings from the larger TECH-N study found that text messaging and community health nursing intervention for adolescents and young adult women with PID decreased subsequent gonorrhea and chlamydia rates, and improved adherence to scheduled follow-up visits.^36^ This sub-analysis of the TECH-N study investigates the text messaging component of the intervention. Specifically, this study (1) analyzes patient responsiveness to an automated text messaging system designed to monitor antibiotic adherence, and (2) measures the reliability of text-reported medication adherence against self-reported antibiotic adherence for adolescents and young women with mild to moderate PID enrolled in the TECH-N study.

## METHODS

### Overview

TECH-N is an active Institutional Review Board (IRB)-approved, RCT (# NCT01640379) with previously described methods.^34,35^ Enrolled patients provided written informed consent, agreeing to be randomized, to participate in a community-based nursing visit if assigned to the intervention arm, and to complete research interviews at 2-weeks, 30-days, and 90-days post-enrollment. Nursing visits were conducted in patients’ homes, except where patients felt it unsafe to do so per protocol. This quality control analysis focused on the fidelity of the text-messaging component of the intervention, which received human subjects’ approval through the Johns Hopkins Institutional Review Board. The analyzed text message and adherence data were collected from September 2012 to June 2015.

### Enrollment, Discharge, and Follow-up

TECH-N research staff recruited patients from the Pediatric and Adult Emergency Departments, General Pediatric primary care clinic, and adolescent medicine/young adult practice within a large urban academic medical center in the mid-Atlantic region of the United States. Eligible patients were 13–25-years-old, English-speaking, female, and residents of the local metropolitan area diagnosed with mild to moderate PID and treated in outpatient settings. Patients who were pregnant, hospitalized for PID, had a language or cognitive barrier, or were re-diagnosed with PID were excluded. Enrolled patients were assigned to the intervention or standard of care (control group) using a computer-generated block randomization sequence.^34,35^ TECH-N provided disposable, pre-paid phones for patients in the intervention arm who lacked mobile phones with text messaging capacity for use during the 90-day duration of the study.

Both the intervention and control groups received 28 tablets of 100 mg doxycycline and were instructed to take two doses or 200 mg daily for 14-days, according to the CDC’s STD treatment guidelines.^7^ Some patients received additional medications (e.g. metronidazole 500 mg twice daily for 14-days) for concomitant treatment of bacterial vaginosis per provider request. At discharge, patients in the control group were instructed to follow-up with their primary care physicians or the institutional Title X-supported Adolescent and Young Adult Clinic within 72-hours of PID diagnosis. The intervention arm received a community-based nursing visit within five days. All patients were scheduled for an outreach research visit immediately following the 14-day treatment period, where patients self-reported their compliance with prescribed antibiotic treatment. Adherence results from the post-treatment interview were uploaded to an online database.

### Text Messaging Intervention

TECH-N staff enrolled patients in the intervention arm in an online health cloud SMS (HCS) system from Reify Health, LLC (http://www.reifyhealth.com)^37^ through which automated reminders and queries were sent. Patients in the intervention arm received a welcome message on the day of enrollment. During the 14-day treatment period, automated text message reminders to take two doses of antibiotics were sent daily at 9 am, and reminders to text back the number of pills taken for that day: “0, 1, or 2” were sent at 7 pm. Simply texting back 0, 1, or 2 was the only step participants had to complete to report adherence.

According to patients’ responses, the HCS system sent text messages to encourage compliance when doses were missed. However, patients were not prompted to respond to encouragement messages. During the 14-day treatment period, fifteen TECH-N text messages (1 welcome and 14 dosage inquiry messages) requested responses. All texts were automated but signed with “TECH-N Nurses” to foster a sense of connection with the study. Text message design was based on earlier focus groups with sexually active women and prior research with similar populations.^28,29^ Text messages were also designed to be interactive and to comply with the automated text messaging system’s delivery capacity. TECH-N intervention messages are listed in Table 1.

### Statistical Analysis

Raw incoming and outgoing text messages data and time stamps from the HCS database were de-identified and downloaded into an Excel (Microsoft Office, 2011) spreadsheet. Messages were sorted according to patient identification numbers, arranged in chronological order, and assigned unique background colors based on message type, i.e. dosage inquiry message, reminder message, patient response (Appendix A). Basic and user-defined excel functions were used to identify messages according to background color or word content for rapid calculation of response rates and sums of text-reported adherence (Appendix B).

### Responsiveness

Individual response rates were calculated based on the number of dosage inquiry messages a patient received and averaged. Percentage of patients responding to a dosage inquiry message was plotted by day (1–14) and evaluated with linear regression to examine attenuation.

### Correlation

The number of pills a patient reported to have remaining in their pill bottle at post-treatment follow-up was downloaded from a separate access database into Excel. This value was subtracted from 28 pills (perfect adherence) to determine self-reported adherence. Zero pills taken was assumed for patients who never texted back. Correlation between total dosage reported *via* text message and total self-reported dosage was evaluated with linear regression for all patients (intention to treat analysis) and for patients with ≥75% response rate (near perfect responders).

### Outcome Measures

The primary outcome measure was average patient responsiveness in percent. Secondary outcome measures were text-reported adherence (sum of daily text reported dosages taken), self-reported adherence, and correlation between text-reported and self-reported adherence. The intention to treat (ITT) analysis of correlation was used with the understanding that non-response may be an inherent problem with texting platforms designed to monitor adherence in clinical trials and clinical settings. Additional correlation analyses were included for participants with response rates of 75% or greater (near perfect responders). These investigations aimed to evaluate the correlation between text message and self-reported medication adherence in a population of participants’ who were committed to texting back.

Participants occasionally responded twice to dosage inquiry messages. Whether participants sent a duplicate text message or responded late to a previous dosage inquiry message was unknown. Duplicate response messages delivered on the same day were ignored to avoid over representing responsiveness or total text reported adherence. Participants who failed to respond to a dosage inquiry message on a given day were assumed to have taken zero pills for that day.

## RESULTS

### Enrollment

One hundred and ninety-two (192) patients were enrolled in TECH-N from September 2012 to June 2015. Ninety-seven of these were randomized into the intervention arm. Three patients were excluded from the intervention arm for either being hospitalized for PID after enrollment, living outside the local metropolitan area, or being previously enrolled in the control group. Two participants were excluded from text message analysis because they enrolled in the HCS late in their treatment period and missed more than five text messages from TECH-N. Another participant was removed from text message evaluation due to continued treatment at the time of data analysis. Ninety-one patients were included in the final analysis. TECH-N maintained a high follow-up rate, with 93.4% of the analyzed intervention group (n=85) returning for the 2-week post-treatment follow-up interview (see attrition in Appendix C). TECH-N also had high follow-up rates at the 1- and 3-month research visits, with 95% of all participants returning for follow-up.

### Demographics

Participants included in this analysis were primarily African American (91%), with mean age of 18.6 (SD=2.2) years. All resided in the Baltimore metropolitan area, and 86.5% were Medicaid-insured and considered low-income. Forty-six (51.6%) had a high school degree or more, 23 (25%) had less than high school education, and 21 (23%) did not provide educational information. These demographics were calculated from 89 participants.

### Message Delivery

HCS logs confirmed that 75 (82%) of the final intervention group (n=91) received the welcome message and 89 (98%) received all 14-dosage inquiry messages. The HCS was programmed to deliver welcome messages to patients in the intervention arm only on the day of their enrollment in the study. Some patients registered in the HCS system after their TECH-N intervention group enrollment date due to phone functionality problems (n=16). These patients (n=16) did not receive the welcome message or any reminder or dosage inquiry messages programmed to deliver on treatment days prior to their HCS activation date. HCS logs confirmed that all other scheduled TECH-N messages were received by these patients. There were no Health Insurance Portability and Accountability Act (HIPAA) violations reported. Cell phone ownership was high, and only five participants required a pre-paid phone for study participation.

### Text Message Responsiveness

**Figure 1.**
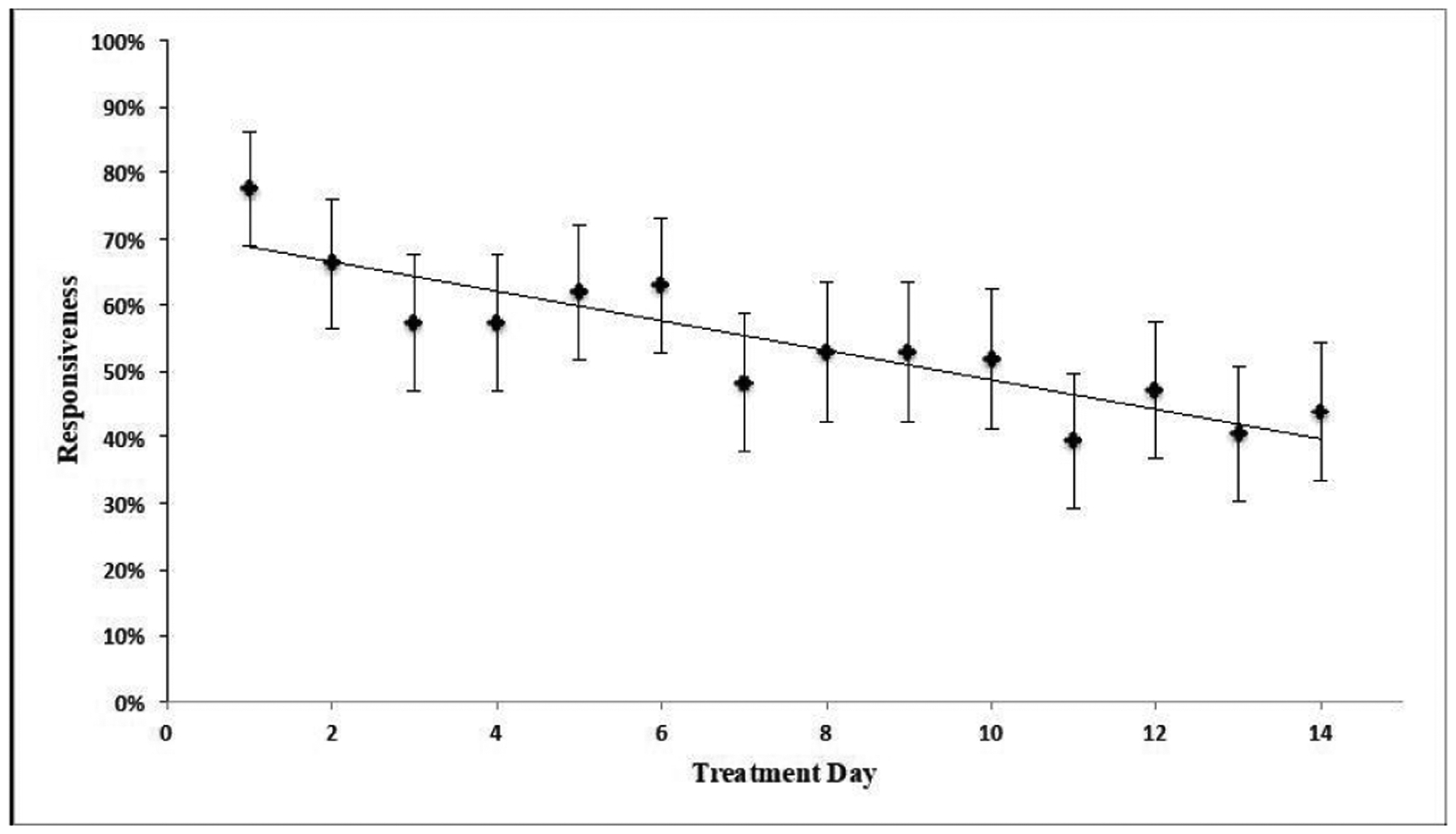
Average Patient Responsiveness to Text Messages by Day and Standard Errors

Fifty-six percent of participants who received the welcome message (n=42 of 75) responded, and 86% (n=78) of intervention participants responded to at least one dosage inquiry message. On average, patients responded to 53% (SD=34%) of dosage inquiry messages received during the treatment period. Responsiveness to dosage inquiry messages attenuated over time, approximating a 2% decrease per day (ß=−0.022, 95% CI −0.03 to −0.015, *p*<0.001) (Figure 1). Average responsiveness to dosage inquiry messages was 61.6% (95% CI, 54.9–68.3%) and 46.9% (95% CI, 43.1–51.0%) for weeks 1 and 2, respectively.

There were 9 perfect responders and 13 participants who never responded. Fifty-nine participants (65%) were actively engaged in interactive texting, responding to more than 40% of received adherence-monitoring messages (Table 2).

There were no statistically significant differences in patient responsiveness to automated text messaging when stratified by age (<20-years *vs*. ≥20-years), education (non-high school graduates *vs*. high school graduates) or insurance type (Medicaid *vs*. private *vs*. no insurance). Of the final intervention group (n=91), 85 participants (93.4%) returned for two-week follow-up.

### Reliability of Text Reported Adherence

**Figure 2.**
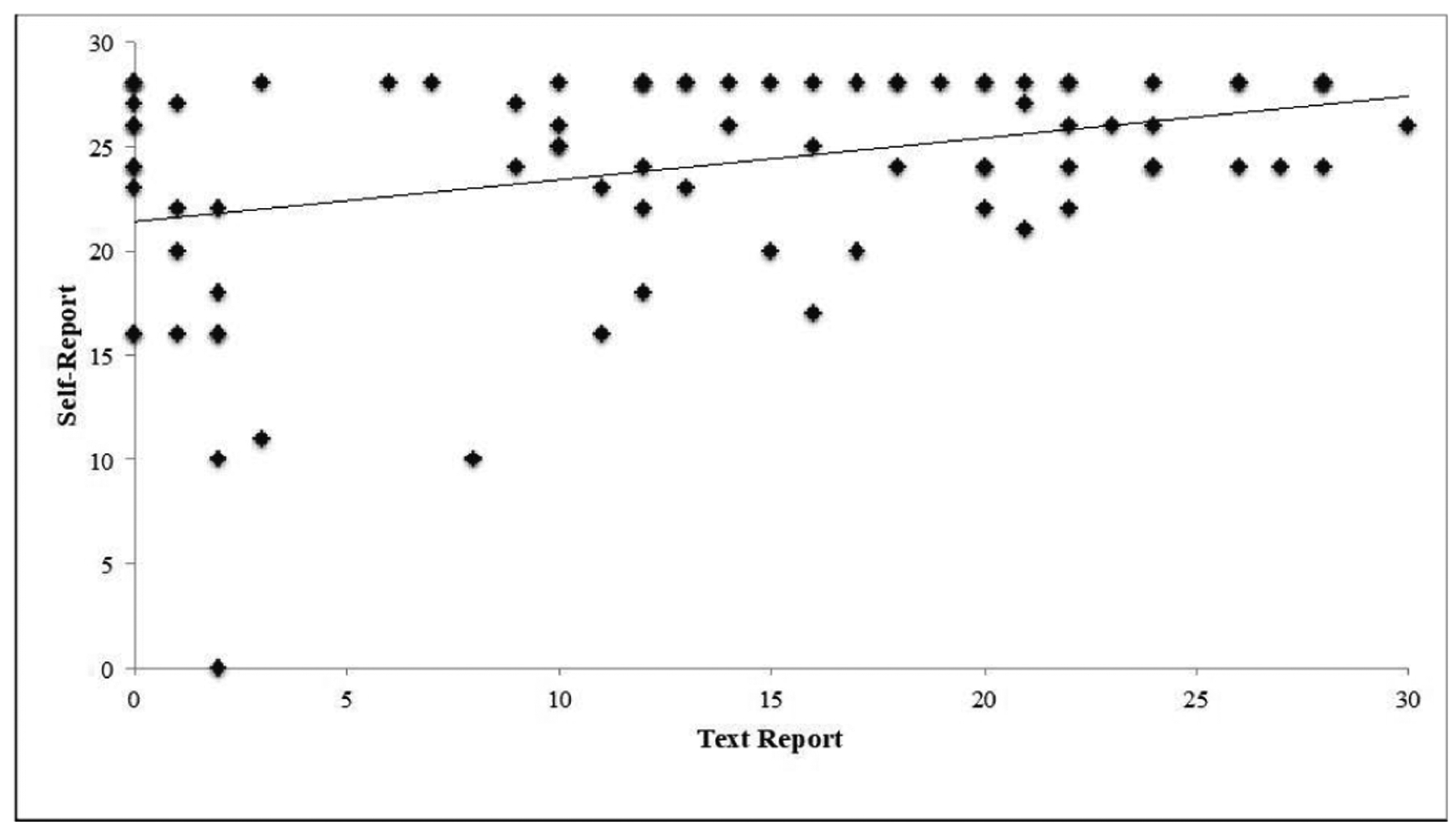
Correlation–Linear Regression of Text-Reported vs. Self-Reported Medication Adherence

When all participants who returned for two-week follow-up were considered (n=85), the intention to treat correlation between text-reported and self-reported medication adherence was positive, but weak (r=0.37, *p*<0.001) (Figure 2). Average total text-reported dosage was 13 (95% CI, 11–15) pills *versus* 24 (95% CI, 23–25) self-reported doses.

A strong correlation (0.82, *p*<0.001) was present when participants who responded to TECH-N messages more than 75% of the time (N=29) were considered. Near perfect responders had an average responsiveness of 89.3% and an average text reported adherence of 22 (95% CI, 20–24) pills *versus* 24 (95% CI, 21–26) self-reported pills.

**Figure 3.**
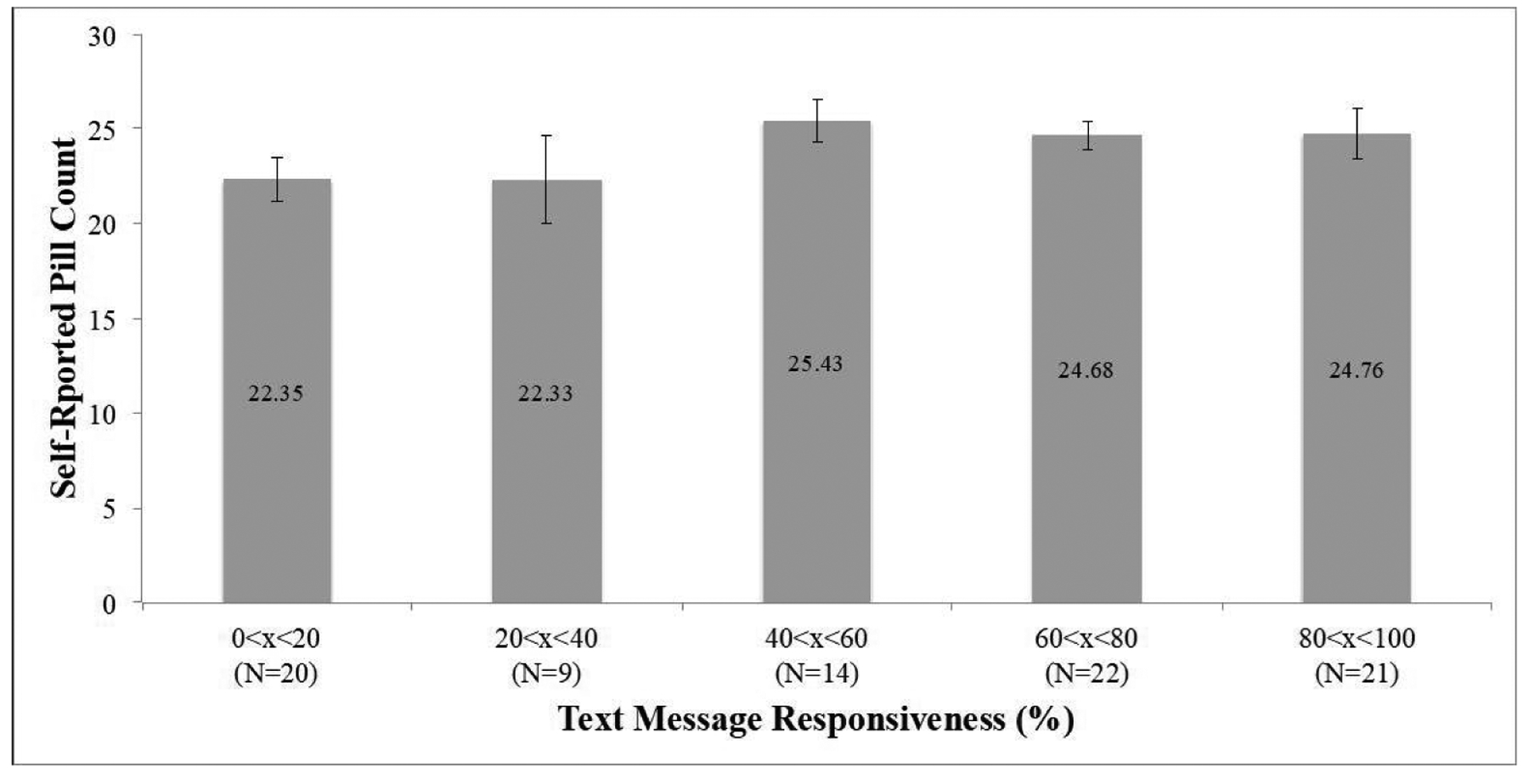
Visual Representation of Self-Reported Pill Count as a Function of Text Message Engagement and Standard Errors

Lower text response rates were not linked to low self-reported adherence (Figure 3). One-way ANOVA (*p*=0.331, significance at *p*<0.05) demonstrated no significant difference in self-reported pill count when intervention patients were categorized by text message engagement. Patients who responded to TECH-N messages 0 to 20% of the time self-reported adherence rates statistically similar to those with 80 to 100% response rates. The average self-reported antibiotic adherence was 86% (24 of 28 recommended pills).

## DISCUSSION

Our research demonstrates that urban adolescents managed for mild to moderate PID with oral antibiotics in outpatient settings are responsive to two-way text messaging communication for additional outpatient clinical support. However, baseline response rates to self-care text-messages varied (mean=53%, SD=34%) and tended to decrease over time. Baseline correlation between text reported and self-reported medication adherence was poor (R=0.37). These findings suggest that response rates and attenuation in responsiveness over time limit the capacity of automated text messaging systems to remotely monitor adolescent’s antibiotic adherence without additional clinical assessment. Enhancing patient responsiveness with two-way texting communication is needed to improve the reliability and adherence monitoring utility of automated text messaging systems for PID support in clinical practice and research.

Responsiveness to self-care text messages was not affected by age, education or economic background. This may be explained by the universality/ubiquity of text messaging. Also, responding to the automated text reminders did not affect patient’s adherence to antibiotic therapy as TECH-N participants self-reported similar medication adherence regardless of text message engagement. Alternatively, simply receiving text message reminders to take antibiotics may have played a role in TECH-N patient’s adherence. Prior studies have shown that 99% of received text messages are opened and that 90% are read within three-minutes of being received.^32^ Evaluating effectiveness of simple text message reminders *versus* two-way text messaging communication is an area for future research.

Enhancing patient’s baseline responsiveness with two-way texting communication to improve the reliability and adherence monitoring utility of automated text messaging systems in clinical practice and research. As most participants completed their treatment months to years before text data was analyzed, a qualitative analysis of adolescents’ non-responsiveness to TECH-N text messages was not performed. However, previous studies demonstrate that adolescents and young adult appreciate receiving health-related text messages from healthcare providers, and that these messages enhance their connection to the treatment team even if they do not respond.^28,38,39^

Text message response rates in this study were similar to prior trials investigating text message interventions for urban adolescents.

Dowshen and colleagues investigated text-messaging intervention for minority HIV-positive adolescents taking anti-retroviral therapy.^30,31^ Participants in this study responded to 61% (SD=30%) of all self-care text messages on average, and the correlation between text-reported and self-reported medication adherence was moderate (R=0.52).^31^ The slightly higher average response rate and correlation in this study might be due to the older age (23-years *vs*. 18-years) and the chronic and socially impactful nature of HIV infection, which requires close interaction with health care providers. Participants also chose the time of day that they wanted to receive text messages and personalized their reminders, which suggest that message personalization may increase text-messaging adherence.

Finally, attenuation in text message response rates overtime suggests that text fatigue may occur. A meta-analysis on text messaging systems for health found that health related text messages delivered at reduced frequency show higher patient compliance than daily text messaging.^32^ Comparing TECH-N results to our previous DepoText Trial supports this finding. DepoText participants received family planning appointment messages once per month and were on average 23% more responsive (76%) to automated text messages^29^ than TECH-N participants (53%), despite similar age, race and background. “Habituation”–human’s tendency to ignore a stimulus delivered frequently-may explain patient’s differed response to daily *vs*. weekly text message reminders.^40^ However, the acute need for PID medication reminder support occurs daily over the 14-days after diagnosis rather than family planning injection behaviors occur every 3-months. Future research investigating patient responsiveness to adherence monitoring messages delivered at shorter *vs*. longer intervals will enhance our understanding of its effect on response rates.

## LIMITATIONS

A notable limitation of our study is the subjective assessment of medication adherence using self-report and its associated risk for reporting bias.^41^ Pill counts were attempted, but participants often did not have pill bottles at follow-up with the outreach staff member. Patients in the intervention arm received a clinical visit from TECH-N nurses three to five-days following PID diagnosis. The influence of nursing visits on medication adherence or study retention were not assessed in this study. Additionally, a few patients were taking medications in addition to doxycycline (i.e., metronidazole) and complex drug regimens have been known to affect adherence.^42^ However, this is the standard of care for PID, and the pill burden of this disorder cannot be overcome. TECH-N reminder messages would still be useful for the twice daily dosing of metronidazole along with doxycycline. .

The problem of handling missing text reported data for correlation analysis was discussed extensively. Single imputation using each participant’s average daily text reported adherence was considered. However, the proportion of missing data (given average response rate of 53%) was too large to employ these methods. We chose to use observed data and present baseline scenarios (all participants no matter their response rate) and best-case scenario (high responders). A 75% response rate or greater was the cut off for near perfect responders as this was a conservative level of text engagement needed to provide a reasonable amount of intervention. Prior research investigating the adherence monitoring capacity of automated text messaging systems for urban youth have evaluated correlation without perfect response rates.^31^ Ultimately, participant non-responsiveness may be an inherent problem with text message interventions in clinical practice.

Finally, this research was conducted at a single urban academic center enrolling primarily low-income African American youth and may have limited generalizability to the general population of PID patient. However, the TECH-N study was executed in a community with significant STI disparities and effectively offered self-care support to vulnerable youth, who are disproportionately affected by PID and often difficult to reach in outpatient settings.

## CONCLUSION

This study takes an important step towards understanding the role of text messaging in supporting adolescents and young adults with PID in outpatient settings and assessing antibiotic adherence for clinical and research purposes. Our findings are in line with prior research demonstrating that adolescents with sexual health concerns are open to additional clinical support *via* text messaging.^28–30,33,39^ It demonstrates that health-related text messaging is a feasible strategy for reaching adolescent and young adult (AYA) patients with PID in outpatient settings. However, baseline response rates to automated text messaging systems make text messaging insufficient as the sole means of monitoring adherence clinical management or research studies in this population.

Future research designed to optimize patient responsiveness to automated text messaging systems may improve automated text messaging systems’ role in antibiotic adherence monitoring. Given the demonstrated attenuation in text message responsiveness overtime and incomplete text reported adherence data, use of additional means to monitor adherence and to support urban youth enrolled in sexual and reproductive health interventions are needed.

## Figures and Tables

**Table 1:** TECH-N Intervention Text Messages

Welcome Message	Thank you for enrolling in the TECH-N study. We will contact you within 24 hours to arrange a follow-up visit. Call us at --- if you don’t receive a call. Text 1 if you got this message. TECH-N Team.
Daily Adherence Reminder	Good morning! Don’t forget to take your medication twice today with a BIG glass of water. TECH-N Nurses
Daily Dosage Inquiry Message	Good evening! How many doses did you take today? Text 0, 1, or 2. TECH-N Nurses.
Sample Intervention Message 1	That’s great! You’re on your way to recovering completely.
Sample Intervention Message 2	One dose is good, but you need to take both doses to make sure your body heals properly.
Sample Sexual Health Message 1	Condoms prevent STDs. Stop by the TECH-N Office if you need some. Call XXX-XXX-XXXX to let us know are coming by. TECH-N Team
Sample Sexual Health Message 2	Birth control is a healthy part of a relationship. Call the Title 10 Clinic at XXX-XXX-XXXX if you need family planning help. TECH-N Team

**Table 2:** Breakdown of Participant’s Level of Text Message Responsiveness (Total N=91)

Patient Responsive to Adherence Monitoring Text Messages	Number of Participants (%)
Never Responded	13 (14%)
Responded At Least Once	78 (86%)
Always Responded	9 (9.8%)
0 to 20% Responsive	22 (24%)
20 to 40% Responsive	10 (11%)
40 to 60% Responsive	14 (15%)
60 to 80% Responsive	23 (25%)
80 to 100% Responsive	22 (24%)
